# Thyroid hormone treatment decreases hepatic glucose production and renal reabsorption of glucose in alloxan‐induced diabetic Wistar rats

**DOI:** 10.14814/phy2.12961

**Published:** 2016-09-21

**Authors:** Silvania da Silva Teixeira, Ana C. Panveloski‐Costa, Aline Carvalho, Fabiana P. Monteiro Schiavon, Any de Castro Ruiz Marque, Raquel S. Campello, Roberto B. Bazotte, Maria T. Nunes

**Affiliations:** ^1^ Department of Physiology and BiophysicsInstitute of Biomedical SciencesUniversity of São PauloSão PauloBrazil; ^2^ Department of Pharmacology and TherapeuticsState University of MaringaMaringaParanaBrazil

**Keywords:** Diabetes Mellitus, gluconeogenesis, liver perfusion, SGLT2, thyroid hormone

## Abstract

The thyroid hormone (TH) plays an important role in glucose metabolism. Recently, we showed that the TH improves glycemia control by decreasing cytokines expression in the adipose tissue and skeletal muscle of alloxan‐induced diabetic rats, which were also shown to present primary hypothyroidism. In this context, this study aims to investigate whether the chronic treatment of diabetic rats with T3 could affect other tissues that are involved in the control of glucose homeostasis, as the liver and kidney. Adult Male Wistar rats were divided into nondiabetic, diabetic, and diabetic treated with T3 (1.5 *μ*g/100 g BW for 4 weeks). Diabetes was induced by alloxan monohydrate (150 mg/kg, BW, i.p.). Animals showing fasting blood glucose levels greater than 250 mg/dL were selected for the study. After treatment, we measured the blood glucose, serum T3, T4, TSH, and insulin concentration, hepatic glucose production by liver perfusion, liver PEPCK, GAPDH, and pAKT expression, as well as urine glucose concentration and renal expression of SGLT2 and GLUT2. T3 reduced blood glucose, hepatic glucose production, liver PEPCK, GAPDH, and pAKT content and the renal expression of SGLT2 and increased glycosuria. Results suggest that the decreased hepatic glucose output and increased glucose excretion induced by T3 treatment are important mechanisms that contribute to reduce serum concentration of glucose, accounting for the improvement of glucose homeostasis control in diabetic rats.

## Introduction

Thyroid hormone (TH) affects several metabolic pathways that are essential for glucose and lipid metabolism (Alkemade 2010). It upregulates genes, like ***Slc2a4***, which encodes GLUT4, an insulin‐regulated facilitative glucose transporter highly expressed in the muscle and adipose tissue, the major sites for postprandial glucose disposal. Moreover, TH regulates genes that encode enzymes that are involved in the utilization of glucose by tissues (Weinstein et al. 1994; Gosteli‐Peter et al. 1996). On the other hand, TH also upregulates genes that encode enzymes that stimulate glycogenolysis and gluconeogenesis, thereby increasing the hepatic glucose production. It is known that glycogenolysis and gluconeogenesis predominate in hyperthyroid states leading to glucose intolerance (Dimitriadis and Raptis 2001).

Type 1 diabetes mellitus (T1DM) occurs as a consequence of autoimmune disease in which pancreatic beta cells are destroyed, resulting in insulin deficiency (Pirot et al. 2008). In this condition, glucose uptake by tissues is impaired and the activity and expression of enzymes that play key roles on the gluconeogenesis and glycogenolysis are increased.

Even though hyperthyroidism seems to worsen metabolic control of T1DM, it can increase the metabolic rate, which is known to increase glucose utilization (Goglia et al. 1999), as well as the ***Slc2a4*** gene expression, increasing glucose transport, effects that could counteract its adverse effects on T1DM. In addition, Brunetto et al. 2012 showed that surgically thyroidectomized rats present reduced GLUT4 protein content, and trafficking to the plasma membrane, as well as a decrease in insulin sensitivity. However, T3 treatment restores these parameters, improving the insulin sensitivity. More recently, Panveloski‐Costa et al. 2016 showed that alloxan‐induced diabetic rats present hypothyroidism and that the T3 treatment improves their glycemia control by decreasing inflammatory cytokines expression.

In the present study, we further investigate possible mechanisms that could account for the improvement of glucose homeostasis control in alloxan‐induced DM rats by evaluating the glucose production, as well as the expression of enzymes involved in gluconeogenesis (PEPCK and GAPDH) in liver, SGLT2 expression in kidney, and urine glucose concentration.

## Material and methods

### Ethics

The experimental protocol was approved by the Animal Care and Ethics Committee of the University of São Paulo and was in agreement with the ethics values in animal research embraced by the National Council for the Control of Animal Experimentation.

### Experimental model

Male Wistar rats weighing 200–250 g were obtained from our own breeding colony and maintained on rat chow and tap water *ad libitum*. They were housed in a room kept at a constant temperature (23 ± 2°C) and on a 12‐h‐light/12‐h‐dark (lights on at 0700 h) schedule. The animals were fasted overnight and rendered diabetic (D) by a single intraperitoneal (ip) injection of freshly prepared solution of alloxan monohydrate (150 mg/kg BW) dissolved in citrate buffer. Nondiabetic control rats (ND) were injected with the same volume of citrate buffer. After 15 days, the glucose level was measured from the tail blood sample. Rats with blood glucose levels below 250 mg/dL were excluded from the study. Diabetic rats were assorted into two groups: nontreated diabetic rats (D), which received i.p. injections of saline for 4 weeks, and treated diabetic rats (DT3), which received i.p. injections of supraphysiological dose of triiodothyronine (1.5 *μ*g/ 100 g BW) for 4 weeks.

### Serum T3, T4 and TSH concentration

Rats were decapitated after anesthesia by sodium pentobarbital (80 mg kg^_1^ BW; Thiopentax, Cristalia, Itapira, SP, Brazil) and blood was collected from the trunk for serum determination of T3, T4, and TSH, as described by Panveloski‐Costa et al. 2016.

### Blood glucose, urine glucose, serum insulin concentration, and glycogen content

Blood glucose was measured with an Optium Xceed blood glucose meter (Abbot Laboratories, IL) from tail blood samples. Serum insulin was determined by rat insulin RIA kit (Millipore, MO). Urine was collected over 24 h from animals kept in metabolic cages and the glucose present in the urine was measured, using an enzymatic Kit (Labtest Diagnostica SA, Brazil). The glycogen was measured from the liver of overnight fasted animals as described by Okamoto et al. 2011.

### Western blotting

Tissue samples were homogenized in buffer containing 20 mmol/L Tris (pH 7.8), 137 mmol/L NaCl, 2.7 mmol/L KCl, 10 mmol/L NaF, 1 mmol/L MgCl2, 0.5 mmol/L Na3VO4, 0.2 mmol/L phenylmethylsulfonyl fluoride, 1 mmol/L EDTA, 5 mmol/L Na pyrophosphate, 10% glycerol, 1% Triton X‐100, 1 *μ*g/mL leupeptin, 1 *μ*g/mL aprotinin. The homogenate was centrifuged at 12,000*g,* 4°C for 20 min and western immunoblotting was performed. To evaluate the AKT phosphorylation, a piece of liver of rats was removed before and after an i.v. administration of regular insulin (10 U). Antibodies were purchased from Cell Signaling (Danvers, MA) to detect phosphorylated AKT at Serine 473, Phosphoenolpyruvate carboxykinase (PEPCK), Glyceraldehyde‐3‐phosphate dehydrogenase (GAPDH) and sodium–glucose cotransporter 2 (SGLT2) and glucose transporter 2 (GLUT2). Ponceau‐S stained membranes were used to control for equal protein loading.

### Liver perfusion

This assay was performed as described by Da Rocha et al. 2013, 2014. Briefly, the animals were anesthetized by an i.p. injection of sodium thiopental (40 mg/kg). The portal vein was cannulated for in situ liver perfusion with Krebs–Henseleit (KH) bicarbonate buffer (pH 7.4) gassed with 95% 02 and 5% C02 and pumped through a temperature‐controlled (37°C) membrane oxygenator. A second cannula was placed into the cava vein to collect the effluent perfusion fluid. The perfusion was performed in an open system (single pass). At the end, the liver was removed and weighed for metabolic calculations and correction of flow rates. After a pre‐infusion period (18 min with KH), glycerol (2 mmol/L) or lactate (2 mmol/L) was dissolved in the KH and infused during 20 min, followed by a postinfusion period (10 min without gluconeogenic substrate) to permit the return of glucose to basal values. The effluent fluid was collected from the inferior cava vein every 5 min for glucose concentration determination. Glucose production during the pre‐infusion period results from glycogenolysis and the variation between the glucose production during and before glycerol and lactate infusion represents the glucose derived from gluconeogenesis. The areas under the curves (AUC) of the pre‐infusion period and infusion period were expressed as *μ*mol/g.

### Statistical analysis

Data are expressed as mean ± SEM. The number of animals utilized in the experiments is shown in the legends of the figures. One‐way analysis of variance (ANOVA) and two‐way ANOVA, followed by Tukey's multiple comparisons test were used for statistical analysis. (GraphPad Prism Software – Version: 6.0 – San Diego, CA).

## Results

### The effect of chronic treatment with thyroid hormone (T3) on serum T3, T4 and TSH concentration of diabetic rat

**Figure 1:**
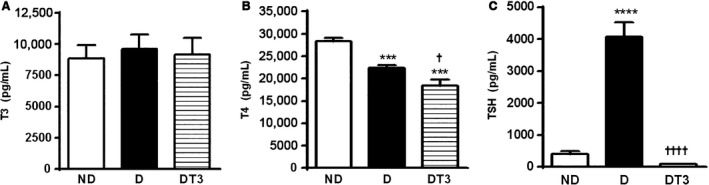
T3, T4 and TSH concentration in nondiabetic control rats (ND), diabetic rat (D) and diabetic rats treated with 1.5 *μ*g/ 100 g BW of thyroid hormone (DT3) for 4 weeks. (A) T3, (B) T4, (C) TSH. Results are expressed as means ± SEM for three independents experiments (*n* = 10). ****P < 0.0001 versus ND group, ***P < 0.001 versus ND group, ††††*P* < 0.0001 versus D group, †*P* < 0.05 versus D group. One‐way ANOVA followed by Tukey's multiple comparisons test were used for statistical analysis.

The diabetic animals had decreased serum T4, unaltered T3 and increased TSH concentrations. In T3‐treated diabetic rats, serum TSH and T4 levels were decreased, whereas T3 concentrations were similar to those of the control (nondiabetic) rats (Fig. 1). These findings corroborate data previously reported by Panveloski‐Costa et al. 2016.

### The effect of chronic treatment with thyroid hormone (T3) on glycemia control of diabetic rat

**Figure 2:**
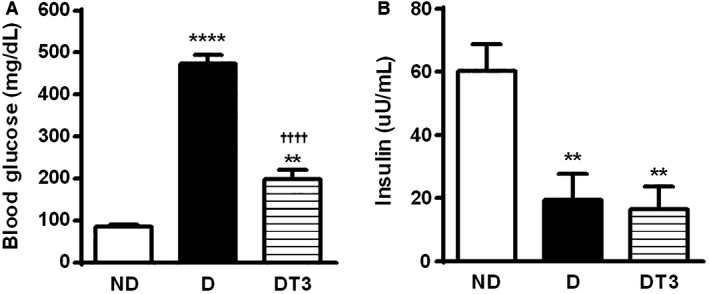
The effect of thyroid hormone treatment on glycaemia control of nondiabetic control rats (ND), diabetic rat (D) and diabetic rats treated with 1.5 *μ*g/ 100 g BW of thyroid hormone (DT3) for 4 weeks. (A) Blood glucose. (B) Serum insulin. Results are expressed as means ± SEM for three independents experiments (*n* = 10). ****P < 0.0001 versus ND group, **P < 0.005 versus ND group, ††††*P* < 0.0001 versus D group. One‐way ANOVA followed by Tukey's multiple comparisons test were used for statistical analysis.

The data presented in Figure 2 show the blood glucose (A) and serum insulin concentration (B) in nondiabetic control rats (ND), diabetic rats (D), and diabetic rats treated with supraphysiological doses of thyroid hormone (DT3) for 4 weeks. As expected, a significant increase on blood glucose values was observed in the diabetic rats. T3 treatment led to a reduction of the diabetic rats glycemia (Fig.2A). Serum insulin concentration of the diabetic rats was decreased, and thyroid hormone treatment did not alter this result (Fig.2B).

### The effect of chronic treatment with thyroid hormone (T3) on gluconeogenesis pathway

**Figure 3:**
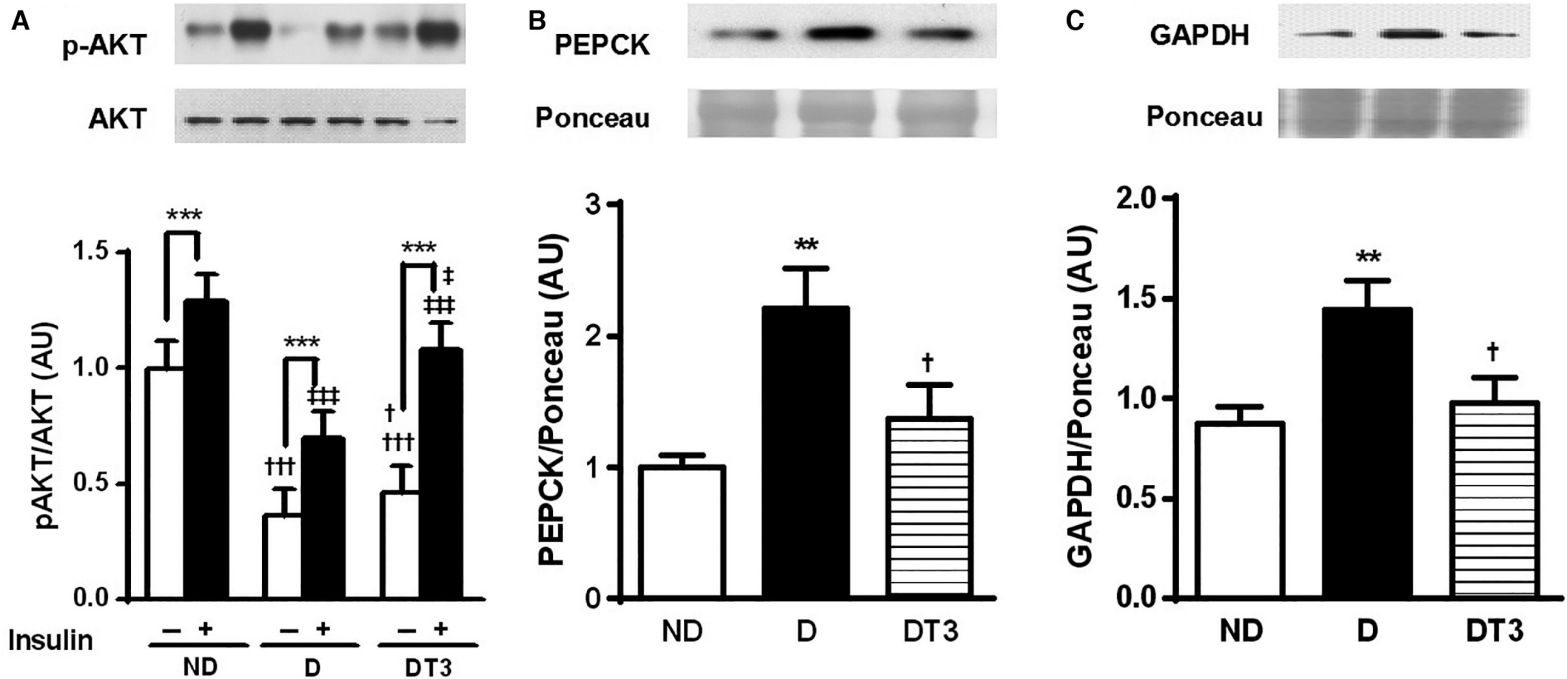
The effect of thyroid hormone treatment on gluconeogenesis pathway of nondiabetic control rats (ND), diabetic rat (D) and diabetic rats treated with 1.5 *μ*g/ 100 g BW of thyroid hormone (DT3) for 4 weeks. (A) Liver Akt phosphorylated at Serine 473. (B) Total liver PEPCK. (C) Total liver GAPDH. Results are expressed as means ± SEM for three independents experiments (*n* = 5). ***P < 0.001 versus (‐) group, **P < 0.005 versus ND group, †††*P* < 0.001 versus ND group (white bars), †*P* < 0.05 versus D group, ‡‡‡*P* < 0.001 versus ND group (black bars), ‡*P* < 0.05 versus D group (black bars). Two‐way ANOVA followed by Tukey's multiple comparisons test were used for statistical analysis.

The data presented in Figure 3 show the AKT phosphorylation in serine before and after insulin administration (A), the protein expression of PEPCK (B) and GAPDH (C) in liver of nondiabetic control rats (ND), diabetic rats (D), and diabetic rats treated with T3 (DT3). The Serine 473 AKT phosphorylation was significantly reduced in diabetic rats both before and after insulin stimulation. The diabetic animals treated with T3 showed an increase in AKT phosphorylation after insulin stimulation, comparable to that found in nondiabetic control rats (ND) (Fig. 3A). The protein expression of PEPCK and GAPDH were increased in diabetic rats, however, after T3 treatment they were significantly reduced (Fig. 3B and C).

### The effect of chronic treatment with T3 on liver glucose production

**Figure 4:**
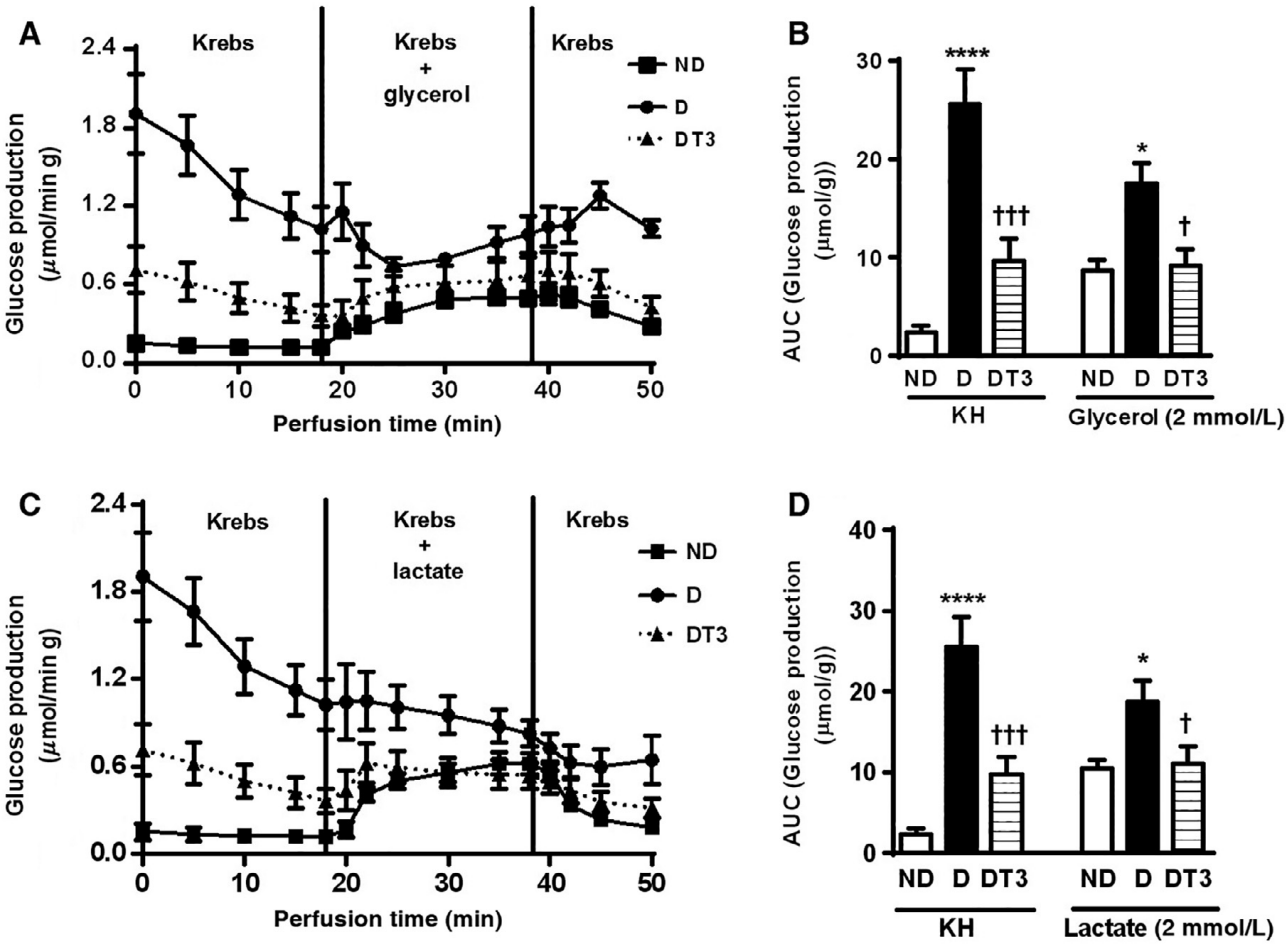
The effect of thyroid hormone treatment on glucose production of nondiabetic control rats (ND) (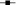),diabetic rat (D) (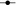) and diabetic rats treated with 1.5 *μ*g/ 100 g BW of thyroid hormone (DT3) (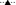) for 4 weeks. (A) Liver perfusion with Glycerol. (B) areas under the curves (AUC) glucose production after Glycerol perfusion. (C) Liver perfusion with Lactate. (D) AUC glucose production after lactate perfusion. Results are expressed as means ± SEM for three independents experiments (*n* = 5). ****P < 0.0001 versus ND group, †††*P* < 0.001 versus D group, *P < 0.05 versus ND group, †*P* < 0.05 versus D group. One‐way ANOVA followed by Tukey's multiple comparisons test were used for statistical analysis.

The Figure 4 shows the liver glucose production after glycerol and lactate administration. Glucose production during the first 18 min represents the glucose derived from endogenous glycogen (glycogenolysis) and the difference between the glucose production during and before glycerol or lactate infusion represents the glucose production from gluconeogenesis. The diabetic rats (D) showed higher hepatic glucose production both before and after glycerol or lactate administration; however it was shown to be decreased before and after the administration of the gluconeogenic stimulus in the diabetic rats treated with T3 (DT3).

### The effect of chronic treatment with thyroid hormone (T3) on glycogen metabolism

**Figure 5:**
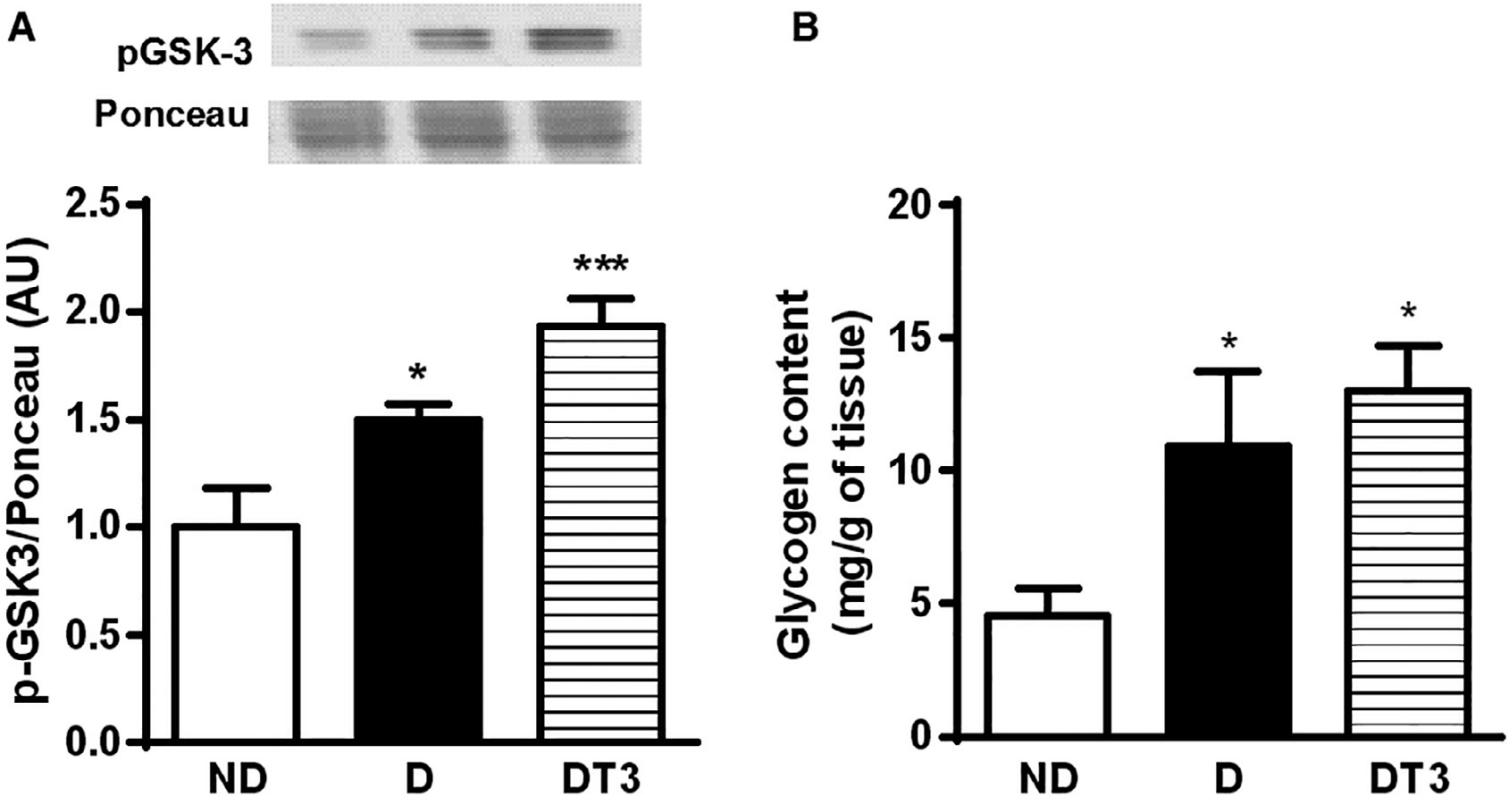
The effect of thyroid hormone treatment on glycogen metabolism of nondiabetic control rats (ND), diabetic rat (D) and diabetic rats treated with 1.5 *μ*g/ 100 g BW of thyroid hormone (DT3) for 4 weeks. (A) Liver phosphorylated GSK‐3 (p‐GSK3) content. (B) Liver glycogen content. Results are expressed as means ± SEM for three independents experiments (*n* = 5). ***P < 0.001 versus ND group, *P < 0.05 versus ND group. One‐way ANOVA followed by Tukey's multiple comparisons test were used for statistical analysis.

Phosphorylated GSK‐3 (p‐GSK3) content (a) and glycogen content (b) in the liver of nondiabetic control rats (ND), diabetic rats (D), and diabetic rats treated with T3 (DT3) are in the Figure 5. The p‐GSK‐3 and glycogen contents were significantly increased in diabetic rats both before and after T3 treatment.

### The effect of chronic treatment with T3 on urine glucose, urine volume, and renal SGLT2 and GLUT2 protein expression

**Figure 6:**
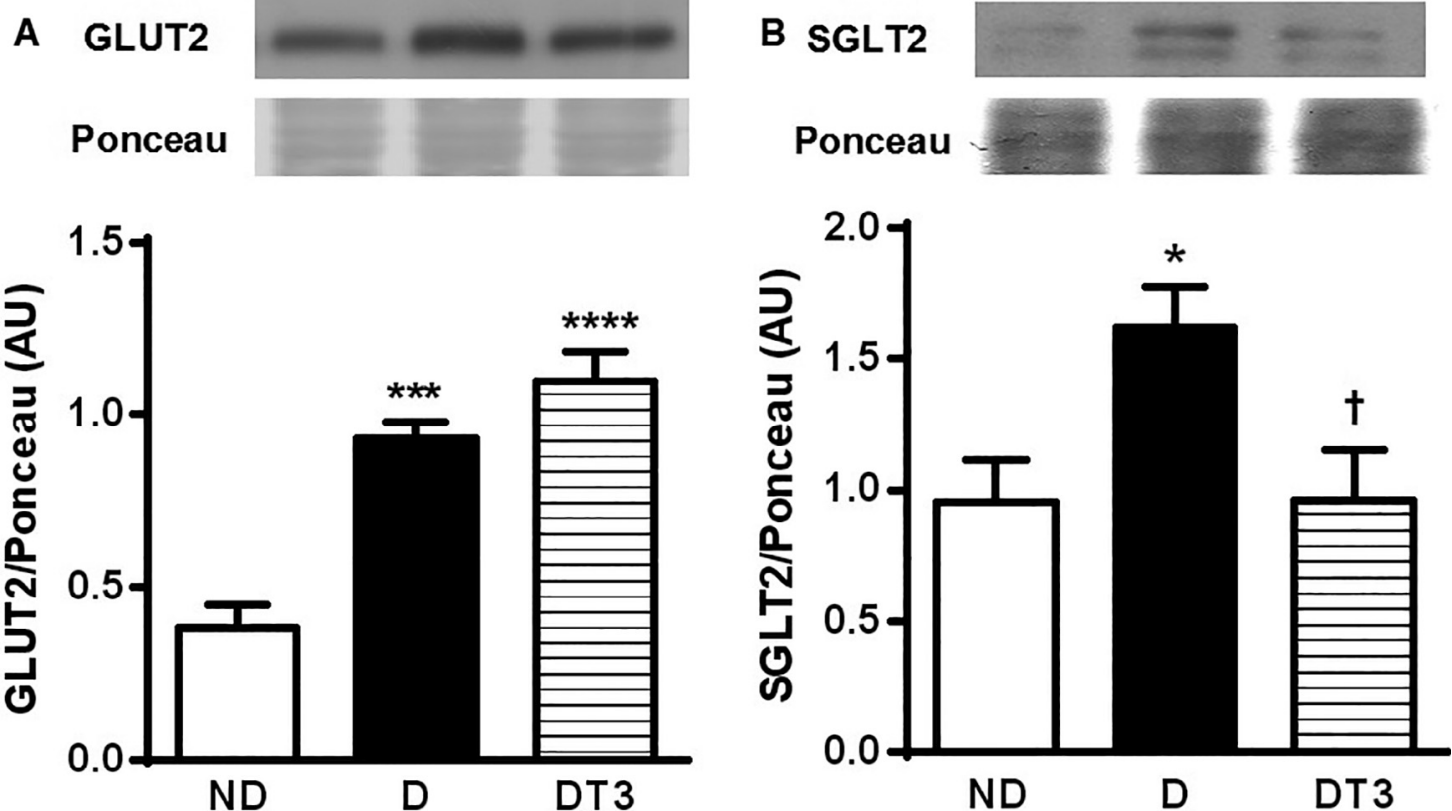
The effect of thyroid hormone treatment on GLUT2 and SGLT2 protein expression of nondiabetic control rats (ND), diabetic rat (D) and diabetic rats treated with 1.5 *μ*g/ 100 g BW of thyroid hormone (DT3) for 4 weeks. (A) Kidney GLUT2. (B) Kidney SGLT2. Results are expressed as means ± SEM for three independents experiments (*n* = 8). ****P < 0.0001 versus ND group, ***P < 0.001 versus ND group, *P < 0.05 versus ND group, †*P* < 0.05 versus D group. One‐way ANOVA followed by Tukey's multiple comparisons test were used for statistical analysis.

**Figure 7:**
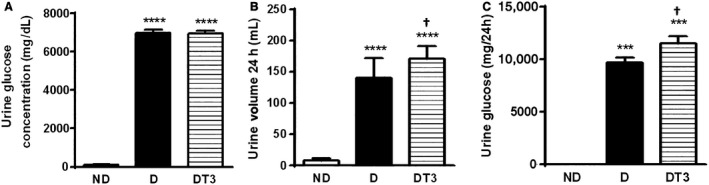
The effect of thyroid hormone treatment on Urine Volume and Urine Glucose of nondiabetic control rats (ND), diabetic rat (D) and diabetic rats treated with 1.5 *μ*g/ 100 g BW of thyroid hormone (DT3) for 4 weeks. (A) Urine glucose concentration in mg/dL, (B) Urine volume/ 24 h, (C) Urine glucose in mg/24 h. Results are expressed as means ± SEM for three independents experiments (*n* = 6). ****P < 0.0001 versus ND group, ***P < 0.001 versus ND group, † *P* < 0.05 versus D group. One‐way ANOVA followed by Tukey's multiple comparisons test were used for statistical analysis.

Figure 6 displays the GLUT2 (A) and SGLT2 (B) protein expression in the kidney and Figure 7 shows the urine glucose concentration in mg/dL (A), urine volume of 24 h (B), and the urine glucose content in 24 h (mg/24 h) (C). The urine volume of 24 h and the urine glucose concentration were increased in both the diabetic (D), and the diabetic treated with T3 (DT3) groups. As expected, the SGLT2 and GLUT2 expression was increased in diabetic rats (D); however SGLT2 content was shown to be decreased and GLUT2 increased in the diabetic group treated withT3 (DT3).

## Discussion

Studies from the literature have shown that chronic treatment with T3 attenuates hyperglycemia and improves insulin sensitivity in db/db mice, which are obese and hyperglycemic (Lin and Sun 2011). These antidiabetic effects of T3 were abolished by the PI3‐kinase inhibitor (LY294002). However, little is known about the effect of chronic treatment with T3 on glucose homeostasis in animal models that mimic DM1. We have shown here that thyroid hormone treatment reduces glycemia, by decreasing gluconeogenesis and renal glucose reabsorption in alloxan‐induced diabetic rats.

Thyroid dysfunction has been associated with diabetes mellitus (Nederstigt et al. 2016). Recently we have demonstrated primary hypothyroidism in alloxan‐induced diabetic rats, and a decrease in the inflammatory cytokines expression on skeletal muscle and white adipose tissue, which could be associated with improvement of insulin sensitivity promoted by T3 treatment (Panveloski‐Costa et al. 2016).

In the present study, diabetic rats treated with T3 presented significant reduction in glycemia (Fig. 2A), even though no change was found in the serum insulin concentration (Fig. 2B). Moreover, alloxan‐induced diabetic rats treated with T3 presented insulin sensitivity similar to that found in nondiabetic control rats, as pointed out by the insulin tolerance test (ITT) (Panveloski‐Costa et al. 2016).

Insulin is an indispensable regulator of the glucose metabolism. Insulin inhibits hepatic glucose production by AKT phosphorylation promoting the inactivation of transcription factor Forkhead box protein O1 (FOXO1), which in turn inhibits target genes transcription, such as PEPCK and glucose‐6‐phosphatase (G6Pase) gene (Barthel et al. 2005). The hypoinsulinemia that occurs in diabetes decreases the AKT phosphorylation and increases the expression of these key proteins of gluconeogenesis (Caton et al. 2009; Cheng and White 2011). These alterations were also observed in the diabetic group of the present study (Fig. 3).

On the other hand, TH stimulates the expression and activity of glycolytic and oxidative enzymes (Short et al. 2001; Moeller et al. 2005), as well as, accelerates the metabolism of glucose, which reduces the intracellular glucose concentration and generates a gradient that favors the entry of glucose into the cells, and increases the expression of glucose transporters on the skeletal muscle and adipose tissue (GLUT4) (Potenza et al. 2009). Moreover, TH also increases the glycogenolysis and gluconeogenesis, which leads to glucose intolerance in hyperthyroid states (Goglia et al. 1999; Dimitriadis and Raptis 2001).

In contrast, our results showed that diabetic rats treated with T3 presented a reduction of PEPCK expression in the liver (Fig. 3B). Moreover, the expression of GAPDH, an essential enzyme in the glycolysis and gluconeogenesis pathways, catalyzing the phosphorylation of the glyceraldehyde‐3‐phosphate to 1.3–biphosphoglycerate (Sakai et al. 2005), was reduced by T3 treatment in alloxan‐induced DM rats (Fig. 3C). Both data suggest that gluconeogenesis is reduced in these animals.

Gluconeogenesis, a metabolic pathway that leads to the production of glucose from noncarbohydrate carbon substrates such as pyruvate, lactate, and glycerol, is one of the main mechanisms to keep blood glucose levels from dropping too low (hypoglycemia). However, the increase in hepatic glucose production is responsible for fasting hyperglycemia in noninsulin‐dependent diabetes mellitus (DeFronzo et al. 1989). Our results corroborated literature data that show that hepatic glucose production by the liver is approximately twofold higher in the diabetic than in the nondiabetic animals (Madison 1969) (Fig. 4). However, our results showed, for the first time, that alloxan‐induced diabetic rats treated with T3 presented hepatic glucose production from lactate and glycerol (gluconeogenesis) similar to that found in nondiabetic control rats (Fig. 4). This reinforces our data showing an increase of pAKT and reduction of PEPCK and GAPDH expression in the liver.

Hepatic glycogen content is mainly regulated by glycemia and insulin action (Shulman et al. 1991; Gerich 1993; Petersen et al. 1998). Glycogen content in the liver of fasted alloxan‐induced diabetic rats was increased as a result of the high blood glucose levels (Fig. 5B) (Friedmann et al. 1963; Gannon and Nuttall 1997). T3 treatment reduced glycemia in alloxan‐induced diabetic rats, however, it did not decrease liver glycogen content (Fig. 5B). GSK‐3 phosphorylation was also significantly increased in diabetic rats both before and after T3 treatment (Fig. 5A). GSK‐3 is active in resting conditions and is inactivated by insulin through phosphorylation. Active GSK‐3 phosphorylates glycogen synthase (GS) and negatively regulates its activity, decreasing glycogen synthesis (Sutherland et al. 1993; Lawrence and Roach 1997). Diabetes condition promoted an increase in liver glycogen content also as a result of the enhanced gluconeogenesis from either glycerol or lactate. Gluconeogenesis leads to formation of glucose‐6‐phosphate that is a precursor of glucose‐1‐phosphate and glycogen (Lehninger et al. 2000). T3 treatment decreased glycemia in alloxan‐induced diabetic rats but did not change liver glycogen content (Fig. 5B). Glucose‐6‐phosphate generation in the liver under untreated diabetic condition was more important to maintain glycogen content than the activity of GSK3. T3 treatment did not promote GSK3 activation (Fig. 5A), reduced gluconeogenesis to control values (Fig. 4) but as mentioned above maintained the high glycogen content possibly due to a reducing effect on hepatic glucose production from glycogen as reported in the presence of KH only (Fig. 4).

The kidney is also involved in the regulation of glucose homeostasis via gluconeogenesis, as well as by reabsorbing glucose from the glomerular filtrate. The renal glucose reabsorption occurs via SGLT2 present in the proximal convoluted tubule and, once reabsorbed, the glucose is released into circulation by the Glucose transporter 2 (GLUT2) located in the basolateral membrane of the proximal tubule (Gerich 2010). The diabetes mellitus increases GLUT2 and SGLT2 expression (Santer and Calado 2010), as observed in diabetic animals in our study (Fig. 6).

Even though the treatment of alloxan‐induced DM rats with T3 decreased the glycemia, the urinary volume and glycosuria in 24 h were higher than those presented in diabetic rats treated with saline (Fig. 7). These data could be explained by a significant reduction of the kidney SGLT2 expression observed in these animals (Fig. 6B). These results show that T3 effects are similar to the SGLT2 inhibitors, which exhibit antidiabetic efficacy in rodent models (Fujimori et al. 2008) and have been proposed as a novel therapeutic strategy for diabetes (Chao and Henry 2010; White 2015).

Taken together, our results point out that T3 is an important regulator of the glucose homeostasis in diabetic states, by decreasing hepatic glucose output and renal glucose reabsorption, suggesting a novel and additional mechanism by which T3 could improve glycemia control and insulin sensitivity in diabetic animals, as described (Panveloski‐Costa et al. 2016).

## Conflict of Interest

The authors have nothing to declare.
